# Neurophysiological and Psychological Consequences of Social Exclusion: The Effects of Cueing In-Group and Out-Group Status

**DOI:** 10.1093/texcom/tgaa057

**Published:** 2020-08-29

**Authors:** Michael Jenkins, Sukhvinder S Obhi

**Affiliations:** Department of Psychology, Neuroscience and Behaviour, McMaster University, Hamilton, Ontario L8S4L8, Canada; Department of Psychology, Neuroscience and Behaviour, McMaster University, Hamilton, Ontario L8S4L8, Canada

**Keywords:** cyberball, event-related potentials, exclusion, group dynamics, P3b

## Abstract

Exclusion by outgroups is often attributed to external factors such as prejudice. Recently, event-related potential studies have demonstrated that subtle cues influence expectations of exclusion, altering the P3b response to inclusion or exclusion. We investigated whether a visual difference between participants and interaction partners could activate expectations of exclusion, indexed by P3b activity, and whether this difference would influence psychological responses to inclusion and exclusion. Participants played a ball-tossing game with two computer-controlled coplayers who were believed to be real. One period involved fair play inclusion while the other involved partial exclusion. Avatars represented participants, with their color matching participant skin tone, and either matching or differing from the color of coplayer avatars. This created the impression that the participant was an ingroup or outgroup member. While ingroup members elicited enhanced P3b activation when receiving the ball during exclusion, outgroup members showed this pattern for both inclusion and exclusion, suggesting that they formed robust a-priori expectations of exclusion. Self-reports indicated that while these expectations were psychologically protective during exclusion, they were detrimental during inclusion. Ultimately, this study reveals that expectations of exclusion can be formed purely based on visual group differences, regardless of the actual minority or majority status of individuals.

## Introduction

Social exclusion has affected virtually every person at least once in their life. Extensive research has contributed to our understanding of the effects of social exclusion on individuals, and has found that these effects are intense and often long-lasting (MacDonald and Leary 2005). The self-reported consequences of social exclusion suggest that exclusion threatens at least four fundamental needs: sense of belonging, self-esteem, sense of control, and sense of meaningful existence (Zadro et al. 2004; Williams 2009). In addition, research has identified a host of negative outcomes that continue after social exclusion, including cognitive impairments (Themanson et al. 2014), behavioral dysfunction (Twenge et al. 2002; Twenge and Baumeister 2004), and motivational changes (Maner et al. 2007; Park and Baumeister 2015), among others.

While the experience of social exclusion will at some point affect all of us, it is more prevalent for some individuals than others. Although social exclusion may occur for many reasons, demographic factors are often involved. In particular, exclusion is often triggered by visual features such as skin tone as these are salient markers that signify membership of specific social groups, some of which are categorized as outgroups (Harrison and Thomas 2009). Categorization of an individual as an outgroup member can set in motion a number of psychological processes that result in prejudice and discrimination toward that individual (Perdue, Dovidio, Gurtman, and Tyler 1990; Gaertner and Dovidio 2009; Ho, Sidanius, Cuddy, and Banaji 2013). It is well established that skin tone modulates the perception of, and behavior toward, observed individuals even when it is task irrelevant (Ito and Urland 2003). Those who appear different to others in their everyday environment, such as members of racial minorities, are more likely to experience chronic social exclusion throughout their lives (Silver 2007).

### Attributing Social Exclusion to Prejudice

The effects of social exclusion are modulated when thought to be due to race, but perhaps not in an immediately intuitive way. For example, Crocker et al. (1991) found that when African American students believed they were being rejected by a White peer, their self-esteem suffered only when they believed they could not be seen. In contrast, when they believed they were visible to their peer, they did not show reductions in self-esteem and self-report measures raise the possibility that suspicion of prejudice could have been a factor. Crocker and colleagues suggested that African American students may have invoked a protective mechanism in conditions where their race was known to the evaluator, which buffered the effects of negative feedback (see also Crocker and Major 1989). Interestingly, this protective mechanism is not only sensitive to negative messages in interpersonal contexts. Evidence also indicates that the (positive) effects of positive feedback and messaging might also be attenuated by members of stigmatized groups because these messages are perceived as an insincere overture to compensate for prejudiced views (Cohen et al. 1999). Interestingly, Mendes et al. (2008) directly compared same-race and different-race interactions among White and Black students and measured responses with accepting and rejecting social feedback. They found that regardless of the race of the participant, different-race rejections were more likely to be attributed to prejudice, and led to more detrimental physiological and performance outcomes including anger. However, Black participants responded less positively to different-race acceptance than did White participants. Taken together, these results suggest that members of stigmatized groups deploy cognitive mechanisms that help buffer the effects of evaluative information emanating from members of another group. This goes hand in hand with the idea of heightened vigilance and threat-sensitivity, and an expectation of rejection, among members of stigmatized groups (Meyer 2003; Fingerhut and Abdou 2017). It has been established that heightened social threat vigilance leads to biases in attention and memory, which highlight the frequency of negative social experiences and downplay the frequency of positive social experiences, and can even lead to behaviors that seek to confirm these biases by perpetuating negative social interactions (see Hawkley and Cacioppo 2011).

Following these studies, Goodwin et al. (2010) directly investigated the mediating role of attributing social exclusion to racism among White and Black individuals. In their study, 614 White and Black adults from a broader noncollege sample engaged in a virtual ball-tossing game called Cyberball (Williams et al. 2000; Williams and Jarvis 2006). This is perhaps the most widely used paradigm for measuring and inducing social exclusion and has proved to be both effective and reliable in this regard (see Hartgerink et al. 2015 for a meta-analysis). As per the standard Cyberball paradigm, Goodwin et al.’s participants believed they were playing an online ball-tossing game with other players represented by avatars, but these players were in fact computer-controlled. The game involved a period of inclusion (“fair play”) in which the participant received the ball one third of the time, and a period of exclusion, in which they received the ball only for the first two throws. A critical difference in Goodwin et al.’s modified paradigm was that the participant and coplayer avatars were represented in full color; participant avatars matched their true skin tone, while the skin tone of the coplayers was manipulated to match, or differ from, the participant. In addition, stereotypical White and Black coplayer names were displayed (e.g., *Bill* vs. *Tyrone*). Following the game, participants’ attributions of their experience to racism were assessed and their sense of fundamental needs fulfillment was recorded. In particular, reflexive and reflective responses to social exclusion were assessed. Reflexive responses are immediate and brief, and appear to be equally strong regardless of contextual or individual differences; ostracism immediately hurts even when it is caused by technical difficulties (Eisenberger et al. 2003) or by a despised outgroup (Gonsalkorale and Williams 2007). Reflective responses occur over time and usually show recovery from the initial harmful effects of exclusion; however, these responses are mediated by contextual and individual differences (see Williams 2007, 2009, for an overview of the distinction between reflexive and reflective reactions to exclusion).

In Goodwin et al.’s (2010) skin tone study, reflexive responses to exclusion by different-race coplayers were more negative for Black participants. However, among both Black and White participants, different-race exclusion was attributed to racism and this impeded reflective recovery from its harmful effects. This study demonstrates that attributions of exclusion to prejudice and their consequences for psychological wellbeing can occur regardless of stigmatized group membership—the role of attributions may in fact be primarily driven by simple visual differences between groups during interactions such as Cyberball.

### Expectations of Social Exclusion

While Goodwin et al. (2010) demonstrate that visual differences between group members influence psychological responses to exclusion, the mechanisms by which this takes place are unclear. That being said, an emerging line of research has begun to uncover the important influence of expectations on subsequent responses to experienced exclusion (see Wesselmann et al. 2017, for an excellent overview). It has been proposed that individuals monitor their environment for exclusionary cues using a “sociometer” (Leary 1999; Leary and Baumeister 2000; Williams 2009). According to sociometer theory, self-esteem is essentially a psychological gauge that tracks the quality of interpersonal relationships. This so-called sociometer constantly monitors the social environment for cues that indicate acceptance or rejection, and is thought to act as a mechanism for avoiding the potentially disastrous consequences of ostracism in the ancestral world (Leary 1999). Some evidence suggests that the perceived accuracy of the sociometer is related to responses to exclusion. For example, Wesselmann et al. (2010) observed more aggressive responses to exclusion when it was unexpected, compared with when it was expected, and this was associated with decreased confidence in the sociometer. In addition, Wirth et al. (2017) found that fundamental needs were more threatened after unexpected compared with expected exclusion, with the same decrease in sociometer confidence also observed in this study. Given that expectations about participation can be modified by both unambiguous cues (e.g., Williams et al. 2000; Maner et al. 2007) and subtle cues (e.g., Wirth et al. 2010; Böckler et al. 2014), it is worth investigating whether visual differences between group members may act as a subtle cue for exclusion, by providing an external factor to which exclusion can be attributed. In this way, it is possible that the participants in Goodwin et al.’s (2010) Cyberball study who attributed exclusion to racism formed exclusionary expectations upon seeing the visual skin tone difference between themselves and the coplayers. Addressing this possibility requires understanding the cognitive mechanisms at play during Cyberball, which would offer insights into how visual differences, attributions, and expectations shape the cognitive appraisal of exclusion as it is experienced.

### The P3b in Social Exclusion Paradigms

While self-report and behavioral measures have been useful for inferring the effects of social exclusion after it has occurred, the EEG technique has proved valuable in observing neural responses to social exclusion while it takes place. In particular, the event-related potential (ERP) technique has highlighted that the P3 component is sensitive to specific inclusionary and exclusionary events, and to the wider context of inclusionary and exclusionary interactions. This component can be subdivided into components that are thought to reflect different levels of attentional allocation; specifically, the P3a, which is generated in frontal regions, and the P3b, which is generated in temporo-parietal regions (Polich 1989; Kok 1997; Rusby et al. 2005; Polich 2007). It is theorized that the P3a is more linked to earlier processing of attended stimuli (Potts et al. 1996; McCarthy et al. 1997; Verbaten et al. 1997), whereas the P3b reflects later interactions between allocation of attentional resources and memory operations (Knight 1996; Squire and Kandel 1999; Brázdil et al. 2001). In particular, the P3b is believed to mark a process of updating working memory representations (Polich 2007, though see Rac-Lubashevsky and Kessler 2016, 2019). While the P3b has often been elicited in response to unexpected or unlikely outcomes (e.g., Kopp et al. 2016), its presence in social exclusion paradigms such as Cyberball probably reflects context-related attentional changes (see Kiat et al. 2018), which relate to the assignment of subjective relevance to specific events.

One of the earliest studies to investigate the P3 response during Cyberball was conducted by Gutz et al. (2011); but see also Crowley et al. 2009). Their study measured the P3a and P3b while participants engaged in a modified game of Cyberball, in which partial exclusion occurred (16% of throws toward the participant, as opposed to 33% during inclusion). Partial, as opposed to full, exclusion is used to ensure that there are sufficient “events” in which participants receive the ball during the wider context of exclusion. They found that both components were sensitive to whether coplayer throws were inclusionary (directed to the participant) or exclusionary (directed to the other coplayer), but that this interacted with the wider context of inclusion or exclusion. Specifically, these components were larger in response to inclusionary throws during exclusionary periods of the game. In addition, this effect was attenuated if exclusion took place first, and larger if exclusion followed initial inclusion. Based on research indicating that the P3 is modulated by stimulus probability in oddball paradigms (Donchin and Coles 1988), and that it marks the allocation of attentional resources and updating of working memory representations (Polich 2007), Gutz et al. (2011) interpreted their results as reflecting a violation of expectations: when exclusion took place, throws toward the participant violated their representation of their participatory status as excluded individuals, resulting in enhanced P3a and P3b activation. Importantly, P3a enhancements were associated with affective processing of exclusion, while P3b enhancements were linked to the perceived intensity of the episode of exclusion.

Since the P3 can be modified by stimulus probability, this raises the possibility that it is not related to any form of social processing, and that its enhancement in response to inclusionary throws within exclusionary contexts is simply driven by the reduced probability of these inclusionary events—receiving the ball during exclusion is less likely to occur, thus rendering it an oddball event (Donchin and Coles 1988). Importantly, Weschke and Niedeggen (2015) ruled out the possibility that P3b enhancements during Cyberball were due to probability, and were in fact sensitive to expectations about social participation. In an elegant design, they independently manipulated the probability of ball throws and the social expectation of receiving the ball. All participants underwent a typical period of inclusion with two coplayers, followed by a period in which the probability of receiving the ball decreased from 50% to 20%. However, one group played this second period with 5 coplayers, instead of 2. For this group, receiving the ball only 20% of the time was in line with their expectations regarding the social nature of the game. Indeed, these participants did not show any P3b enhancements when receiving the ball, despite the low likelihood of this event. As such, Weschke and Niedeggen proposed that the P3b effect observed within Cyberball paradigms is primarily driven by a cognitive process that is sensitive to expectancy violations, and is sensitive to high-level social expectations related to exclusion.

Given the link that Gutz et al. (2011) propose between the P3 and expectations, and the fact that this can be measured within an ERP Cyberball paradigm, the question arises as to whether the formation of exclusionary expectations caused by external factors (rather than the actual experience of inclusion or exclusion) can influence the P3 response during Cyberball. Though this has not yet been studied in detail, some research provides convincing evidence that this is the case. For example, Gutz et al. (2015) ran a similar Cyberball study on participants with borderline personality disorder (BPD), who tend to express interpersonal dysfunction and are thought to engage in biased processing of social threat information (Clark and Wells 1995; Arntz et al. 1999). While healthy controls showed the established pattern of enhanced P3b responses to inclusionary throws during exclusion (but not inclusion), BPD participants showed this pattern during both inclusion and exclusion, suggesting that the representation of participatory status formed by BPD participants was one of exclusion, regardless of actual experience. This was mirrored in self-report data, which highlighted increased threatening of needs during inclusion, and lower perceived proportion of throws received; that is, even when BPD participants received the ball equally as often as their coplayers, they reported receiving it less often. This study demonstrates that different psychological states can result in negatively biased perceptions of social interactions, which in turn influence P3b activation patterns.

The relationship between exclusionary expectations and the P3b has also been demonstrated outside of the Cyberball paradigm, and in the context of stigmatized group status. Kiat et al. (2017) employed a simple task in which White and Black participants made choices that were either neutral (“Dog” vs. “Cat”) or stereotyped with regard to Black American culture (“Hip Hop” vs. “Rock and Roll”). Following their choice, an image was presented in which their avatar was seated at a lunchroom table either with their “best friends” (inclusion) or alone (exclusion). ERP results revealed an enhanced P3b response to inclusion images only after Black participants made stereotyped choices, suggesting that these choices provided a cue for attributing exclusion to their race. Since this resulted in exclusionary expectations, subsequent images of inclusion presented a discrepancy with participants’ representation of their participatory status, possibly triggering mechanisms involved in updating these representations in memory, as indexed by the P3b component.

### The Current Study

When taken together, these ERP studies suggest that subtle cues can enhance expectations of exclusion and result in modified neural responses to the experience of exclusion, and inclusion, as it takes place in real time. Considering previous self-report Cyberball studies such as Goodwin et al. (2010), in which attributing exclusion to visual group differences altered the effects of exclusion, it is possible that such visual differences would also enhance exclusionary expectations, which could be measured using the P3b. To address this, we employed a modified Cyberball paradigm in which participants were represented as avatars with white or brown skin tones and believed they were playing with other players, who were in fact computer-controlled and were represented with avatars whose skin tone either matched or differed from the participant. ERPs were measured in response to inclusionary and exclusionary throws, during wider periods of inclusion and exclusion, with a focus on the P3a and P3b components. After the game was complete, we assessed fundamental needs through self-report measures.

We predicted that participants made to appear visually different (in terms of skin tone) from their coplayers would show enhanced P3b responses to inclusionary throws relative to exclusionary throws during both periods of inclusion and exclusion, while those made to appear similar to their coplayers would only show this pattern during exclusion. This would suggest that skin tone differences act as a subtle, task-irrelevant cue that creates exclusionary expectations. Since previous research suggests that this would specifically influence processes related to subjective relevance and working memory representations (Gutz et al. 2015; Kiat et al. 2017), we predicted that skin tone differences would not modulate the pattern of P3a activation.

With regard to the psychological outcomes resulting from exclusion, previous findings are not straightforward. While behavioral (Crocker et al. 1993; Crocker 1999; Major et al. 2003) and neuroimaging research (Masten et al. 2011) suggests that attributing exclusion to factors such as skin tone can act as a protective buffer, reducing reactivity and thereby limiting its harmful effects, other studies have demonstrated clearly negative outcomes, including anger (Mendes et al. 2008) and slowed recovery of needs fulfillment (Goodwin et al. 2010). Overall, there is a consensus that intergroup rejection evokes external negative reactions such as anger, while intragroup rejection results in internalized reactions such as self-blame, which has implications for self-esteem (see Crocker and Major 1989). In view of these considerations, we predicted that needs fulfillment as measured immediately after the Cyberball game would be less harmed by exclusion from different skin tone coplayers relative to same skin tone coplayers.

## Materials and Methods

### Participants

Forty-nine McMaster university students were recruited for the experiment in exchange for course credits. Because McMaster University contains large populations of White and South Asian individuals, these groups were the focus of our skin tone manipulation. Prescreening ensured that only participants who self-reported as Caucasian or South Asian were recruited. One participant was excluded from analysis due to excessively noisy EEG activity (more than 15% of trials removed during artifact rejection). The remaining 48 participants consisted of 28 White-skinned (20 female and 8 male) and 20 Brown-skinned (12 female and 8 male) individuals aged between 18 and 21 (mean age 19.13). Sample size was determined based on a simulation-based power analysis; this sample size was estimated to achieve 81% power to observe a three-way interaction between the factors possession, social context, and skin tone with a “large” effect size (${\eta}_p^2$) of 0.14. Informed consent was provided by all participants in accordance with the Declaration of Helsinki (1991, p. 1194) and they were debriefed at the end of the experiment, at which point the true nature of the study was revealed. Ethical approval was granted by the McMaster Research Ethics Board (MREB, certificate # 0669).

### Procedure

Upon arrival, participants were informed that the experiment was investigating the neural correlates of visual imagination during interactions, and that two other participants had also recently arrived and would be tested simultaneously in separate testing rooms. In fact, there were no other participants being tested. After providing informed consent, participants were given the Vividness of Visual Imagery Questionnaire (VVIQ, Marks 1973); this has been administered in several other studies using the Cyberball paradigm (e.g., Gutz et al. 2011, 2015) and acts to provide a false cover story to prevent participants’ awareness of the true nature of the study. During completion of the VVIQ, the experimenter entered the participant’s age, name, and gender (Male or Female) as provided by the participant, and discretely recorded the participant’s skin tone (White or Brown) and hair color (Blonde, Brown, Black, or Red). Participants were randomly and discretely assigned to one of two skin tone conditions (same or different).

This program was run on MATLAB 9.2 (R2017a) (Mathworks, Inc., MATLAB 2017), using the Cogent 2000 Toolbox (www.vislab.ucl.ac.uk/Cogent/) to display stimuli on a gray background. The following instructions were provided to participants on the screen: “In this experiment, you will be playing an online ball game with two other participants. At different times, you will be asked to imagine playing the ball game in a given setting. We have generated a simple avatar to represent you during this game. This is you!.” Alongside these instructions appeared a blank face that was selected from a set of 16 images composed of each combination of gender, skin tone, and hair color. The selected face matched as closely as possible the properties of the participant. Below this face, some additional text also appeared on screen: “The other two players will have avatars representing them. We want you to imagine really playing the ball game with these people.” Participants were reminded to minimize eye movements and fixate on a point in the center of the screen throughout the experiment. Following instructions, a waiting screen was displayed in which the participant was told how many players were ready; this amount was randomly selected between 1 and 3. After a random of interval of 5–30 s, the number was increased until all three players were ostensibly ready. A practice session then began, with three face images on-screen: The participant’s avatar (as presented during instructions) appeared in the bottom-center of the screen, while the two coplayer avatars appeared in the top-left and top-right corners. These were randomly generated from the same set of images as the participant’s avatar, on the basis of their details and their assigned condition. Specifically, the gender of all avatars was always the same (3 males or 3 females), and the skin tone of the coplayer avatars matched that of each other, and either matched or differed from that of the participant depending on their skin tone condition assignment. Coplayers were assigned names which appeared under their avatars, based on the participants’ ethnicity, gender, and skin tone condition: White-skinned coplayers had the names “Amy” and “Claire” for females, and “Jake” and “Connor” for males. Brown-skinned coplayers had the names “Miryam” and “Prisha” for females, and “Vihaan” and “Aarav” for males. Participants did not see their own name on the screen, but were told that the other players would see their name. In addition, a black and white soccer ball was drawn on-screen. Initially, the ball randomly appeared next to either of the three players. From this point onwards, the program waited indefinitely for the participant to press either the left or right arrow key on the keyboard whenever the ball was adjacent to the participant. After a key response, the ball was drawn slightly larger at the center of the screen for 500 ms to give an impression of rising into the air and was then drawn at the original size adjacent to the left or right coplayer, depending on the key press. Once the ball was in possession of a coplayer, following a random delay of 500–2500 ms, the ball was “thrown” (using identical animations as the participant throws) either to the participant or to the other coplayer. An initial practice phase included 10 coplayer throws, with 5 directed to the player.

**Figure 1:**
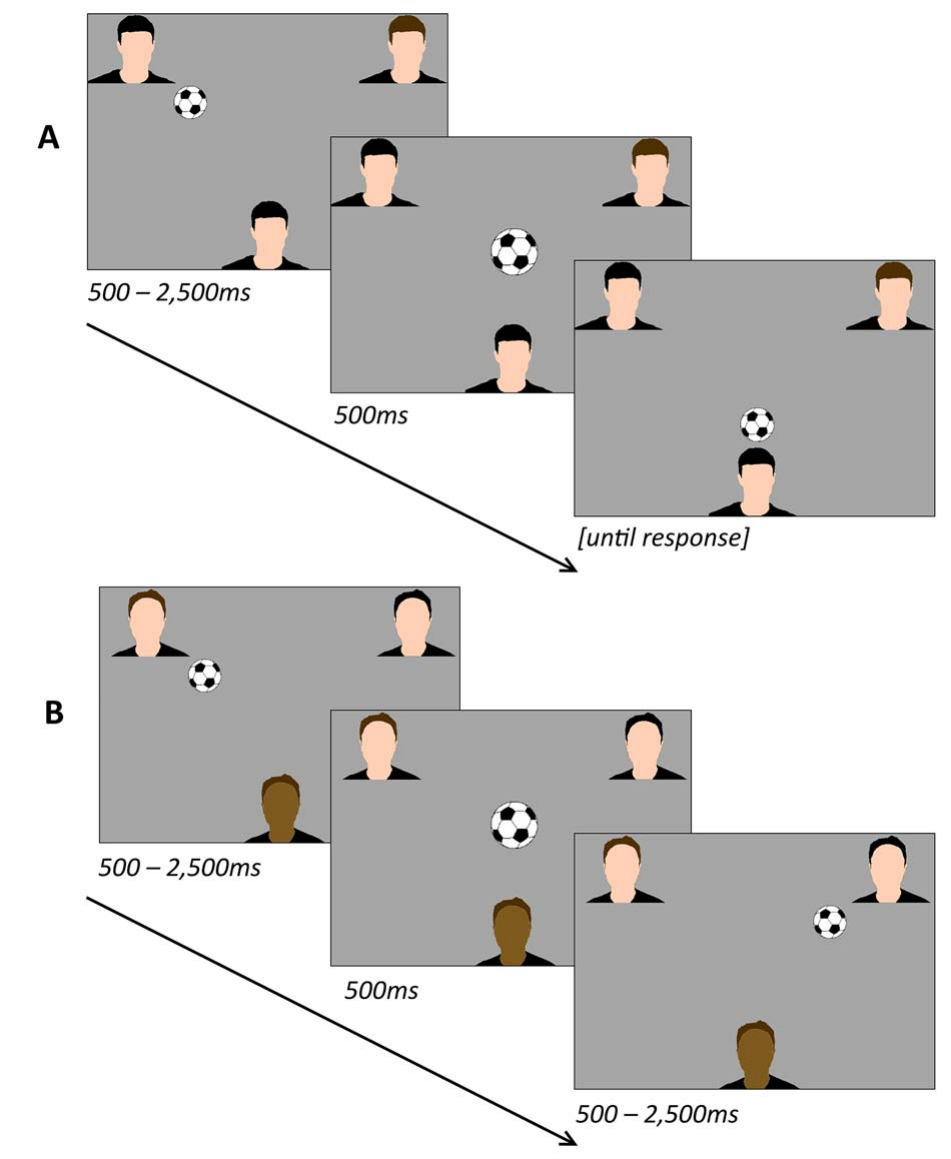
Schematic of events within the Cyberball game. During each throw, the ball appeared in the center of the screen briefly before appearing at the location of a player. (*A*) A “self” event, in which a White-skinned male participant (bottom avatar) assigned to the same condition receives the ball from a coplayer (top avatars). (*B*) An “Other” event, in which a Brown-skinned female participant assigned to the different condition observes one coplayer throw the ball to the other coplayer. Each skin tone condition contained the same number of White (14) and Brown (10) participants.

After the practice, new instructions appeared stating that participants would be presented with an image of a scene at regular intervals throughout the task. Following these instructions, the same waiting screen appeared as before; once all players were supposedly ready, the main task began with an image of a field or a gymnasium, alongside the text “While playing the next ball game, try to imagine playing it here.” After 10 s, the ball game began exactly as it did during the practice session. However, the program now used a predetermined trial specification to determine whether each coplayer throw would be directed either at the player (“self” event) or at the other coplayer (“other” event). Two trial specifications were used, corresponding to periods of inclusion versus exclusion. Periods of inclusion consisted of 60 coplayer throws, with 30 (50%) directed at the participant. For periods of exclusion, it consisted of 75 coplayer throws, with 15 (20%) directed at the participant. Because each throw directed at the participant led to an additional throw by the participant, both trial specifications resulted in 90 total throws, lasting ~2.5 min. The order of these periods was counterbalanced across participants (Fig. 1).

For each period, the game was separated into four blocks, each using the same trial specification but shuffling the order of trials each time. During exclusion blocks, shuffling was constrained so that “Self” events never repeated sequentially, to avoid the illusion of reinclusion. The image shown before each block alternated between the field and gymnasium images. In total, the entire game resulted in 120 self events and 120 other events during inclusion, and 60 self events and 240 other events during exclusion, for a total of 540 events. Each period lasted ~10 min, with a 60-s break between periods. Depending on their order condition assignment, participants either underwent inclusion or exclusion first.

EEG trigger codes were used to mark the moment that each coplayer throw reached its target. Because the ball appeared as static images, this moment was the earliest point in time that participants could be aware of whether the ball was thrown toward them (“self” event) or toward the other coplayer (“other” event). Trigger codes distinguished between the two within-subjects factors: separate codes were used for self and other events (possession factor), and for inclusion and exclusion periods (social context factor), resulting in four different event conditions.

Upon completion of the Cyberball game, the program closed and participants were given two instances of the Need-Threat Questionnaire (NTQ, Van Beest and Williams 2006); each corresponded to the separate periods of the game (referred to as “first half” and “second half”) and asked participants to consider how they felt during each period when responding. After the EEG cap was removed, they were given the Rejection Sensitivity Questionnaire (RSQ, Downey and Feldman 1996). Due to a change in protocol part way through the period of data collection, half of participants (those collected during the latter half of data collection, *N* = 24) were given three additional questions to assess the extent to which exclusion was attributed to racial prejudice. These were identical to the attribution questions used by Goodwin et al. (2010), asking participants to rate from 1 to 5 the extent to which they believed (1) they had been treated as they were due to their ethnicity, (2) they had been discriminated against, and (3) that the coplayers were racist. Finally, all participants were given a debrief form along with the opportunity to withdraw after discovering the true nature of the study.

### E‌EG Data Collection

EEG was recorded using a 64 channel Neuroscan Quik-Cap, using a 36-channel montage (FP1, FP2, F7, F3, Fz, F4, F8, FT7, FC3, FCz, FC4, FT8, T7, C3, Cz, C4, T8, TP7, CP3, CPz, CP4, TP8, P7, P3, Pz, P4, P8, PO7, PO3, PO4, PO8, O1, Oz, O2). The ground electrode was positioned between FPz and Fz, and the reference electrode was positioned between Cz and CPz. Impedances were kept below 10 kΩ prior to data collection. Data were online referenced to the reference electrode and sampled at 1000 Hz. After data collection, data were transferred to the EEGLab plugin (Delorme and Makeig 2004) and rereferenced offline to the average of two electrodes placed on the mastoids and initially bandpass filtered with an FIR filter (using EEGLab’s “pop_eegfiltnew()” function) between 1 and 20 Hz for the purposes of independent component analysis (ICA). Data were then epoched based on each of the four event conditions using the ERPLab plugin (Lopez-Calderon and Luck 2014). ICA was then run on each dataset using EEGLAB’s SOBI function (Second-Order Blind Identification; Sahonero-Alvarez and Calderon 2017). The derived ICA weights were then transferred to the dataset using a more conservative bandpass filter of 0.1–40 Hz. The MARA plugin (multiple artifact rejection algorithm; Winkler et al. 2011, 2014) was then used to automatically classify and subsequently remove components classified as artifacts. Finally, all remaining trials that contained ±100 μV waveforms were removed. In total, no more than 6.67% of trials were removed per participant.

Using the ERPLab plugin, subject ERPs were created by averaging epochs within each event condition. To ensure a comparable signal-to-noise ratio (SNR) across event conditions, trials from all conditions except for self-exclusion were randomly removed until 60 events remained. While mean amplitude analysis is not considered to substantially benefit from equal SNR (e.g., Luck 2005, 2012; Clayson, Baldwin, and Larson 2013), we chose this method to facilitate other forms of ERP analysis if required. To further improve comparability, “self” events that were immediately preceded by other “self” events (which was possible only during the Inclusion period) were removed from analysis.

To determine suitable electrode sites for mean amplitude analysis, grand average positive peak latencies (averaged across all events and all participants) were detected at the 15 central electrode sites (F3, Fz, F4, FC3, FCz, FC4, C3, Cz, C4, CP3, CPz, CP4, P3, Pz, P4) between 200 and 300 ms poststimulus for the P3a and between 300 and 400 ms for the P3b, and mean amplitudes were measured across time windows extending 40 ms before and after these latencies. The highest mean amplitude extending from the peak in the 200 and 300 ms time window was observed at electrode Cz (6.55 μV), and in the 300 and 400 ms time window at electrode CPz (5.48 μV). For these two electrode sites, positive peak latencies were detected separately for each combination of possession, social context and skin tone, and mean amplitudes were again measured extending ±40 ms from these latencies (The same analyses were conducted at electrodes FCz (for the P3a) and Pz (for the P3b), as these sites have often been used to assess P3a/P3b activity (Themanson et al. 2015). In addition to peak-defined time-windows, we calculated mean amplitudes using fixed time windows of 230–310 ms for the P3a, and 310–390 ms for the P3b, as these have also been employed in previous research (Gutz et al. 2011, 2015). All of these analyses produced virtually identical results.).

P3a and P3b mean amplitudes were separately entered into a 2 × 2 × 2 repeated-measures ANOVA with the within-subjects factors possession (self, other) and social context (inclusion, exclusion), and the between-subjects factor skin tone (same, different). Follow-up *t*-tests were conducted to assess interactions, with the Holm-Bonferroni correction applied where multiple familywise comparisons were made.

## Results

### ERP Results

#### P3a Component

For the P3a component (at electrode Cz), the ANOVA revealed a main effect of social context (*F*(1,44) = 4.13, *P* = 0.048, ${\eta}_p^2$ = 0.09), indicating slightly larger (more positive) P3a mean amplitudes during exclusion (6.96 μV) than inclusion (6.36 μV, mean difference = 0.60 μV). A main effect of possession (*F*(1,44) = 27.25, *P* < 0.0001, ${\eta}_p^2$ = 0.38) indicated larger P3a activity in response to self events (7.79 μV) versus other events (5.53 μV, mean difference = 2.26 μV). This was accompanied by an interaction between possession and social context (*F*(1,44) = 90.99, *P* < 0.0001, ${\eta}_p^2$ = 0.67). Follow-up *t*-tests revealed no difference between self and other events during inclusion (mean difference = 0.40 μV, *t*(75) = 0.77, *P* = 0.44), but significantly larger responses to self (9.42 μV) versus other (4.49 μV) events during exclusion (mean difference = 4.93 μV, *t*(75) = 9.55, *P* < 0.0001). There was no interaction between possession, social context, and skin tone (*F*(1,44) = 0.04, *P* = 0.84), and no other main effects or interactions were observed (all *F*(1,44) < 2.18, all *P* > 0.14).

#### P3b Component

For the P3b component (at electrode CPz), the ANOVA revealed a trend toward a main effect of social context (*F*(1,44) = 3.97, *P* = 0.052, ${\eta}_p^2$ = 0.08), in which P3b amplitudes were slightly larger during exclusion (5.90 μV) than during inclusion (5.37 μV, mean difference = 0.53 μV). A main effect of possession (*F*(1,44) = 41.96, *P* < 0.0001, ${\eta}_p^2$ = 0.48) indicated larger P3b activity for self events (7.30 μV) than for other events (3.97 μV, mean difference = 3.34 μV). This was accompanied by an interaction between possession and social context (*F*(1,44) = 104.93, *P* < 0.0001, ${\eta}_p^2$ = 0.70). Follow-up *t*-tests revealed no difference between self and other events during inclusion (mean difference = 0.51 μV, *t*(67) = 0.88, *P* = 0.38), but significantly larger responses to self (8.98 μV) versus other (2.82 μV) events during exclusion (mean difference = 6.16 μV, *t*(67) = 10.54, *P* < 0.0001). In addition, social context interacted with distinctiveness (*F*(1,44) = 6.83, *P* = 0.01, ${\eta}_p^2$ = 0.13), indicating significantly larger responses during exclusion (6.23 μV) than inclusion (5.01 μV) for those in the same condition (mean difference = 1.22 μV, *t*(44) = 3.26, *P* < 0.01), but no difference for those in the different condition (mean difference = 0.16 μV, *t*(44) = 0.44, *P* = 0.66).

**Figure 2:**
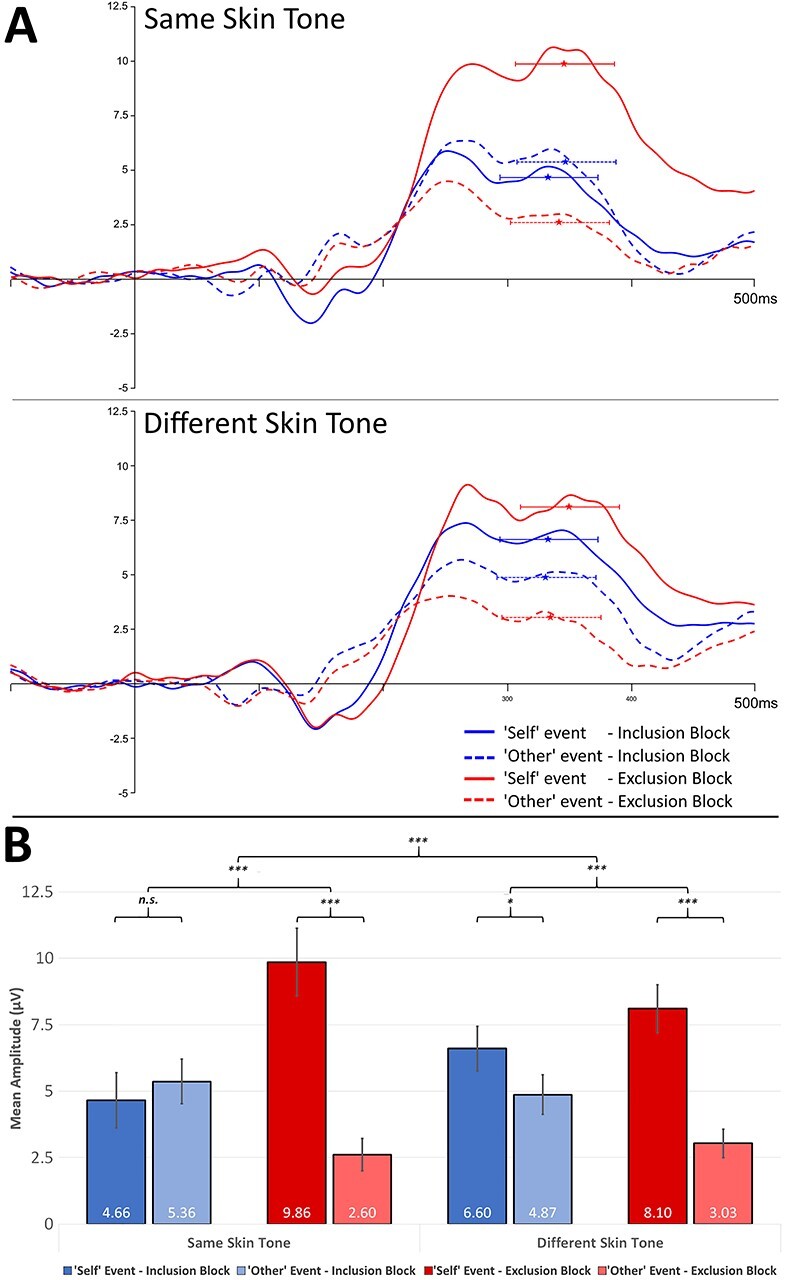
(*A*) Grand-averaged ERP waveforms elicited at electrode CPz by “self” and “other” events, during periods of inclusion and exclusion, shown separately for participants assigned to the same and different conditions. Stars represent peak latencies and mean amplitudes for each trial condition, with horizontal bars representing the peak-defined time window used for analysis of P3b mean amplitudes (±40 ms from peak latency). (*B*) P3b mean amplitudes in each condition (error bars represent SEM). “Self” versus “Other” events are compared separately for inclusion and exclusion and separately for participants in each skin tone condition. In the different condition, participants showed significantly larger P3b amplitudes to self versus other events during inclusion as well as exclusion, qualifying a three-way interaction between social context, possession, and skin tone.

Critically, a three-way interaction between possession, social context, and skin tone was observed for the P3b (*F*(1,44) = 17.53, *P* < 0.001, ${\eta}_p^2$ = 0.28). To assess the nature of this interaction, two separate repeated-measures ANOVAs were run with the within-subjects factors possession and social context, separately for each skin tone condition. For participants in the same condition, an interaction between the two factors (*F*(1,23) = 65.04, *P* < 0.0001, ${\eta}_p^2$ = 0.74) revealed larger amplitudes for self (9.86 μV) versus other (2.60 μV) events during exclusion (mean difference = 7.25 μV, *t*(41) = 8.40, *P* < 0.0001), but not during inclusion (mean difference = 0.71 μV, *t*(41) = 0.82, *P* = 0.42). For participants in the different condition, the same interaction was observed (*F*(1,23) = 38.48, *P* < 0.0001, ${\eta}_p^2$ = 0.63), but was significantly less pronounced. Amplitudes were larger in response to self (8.10 μV) events versus other (3.03 μV) during exclusion (mean difference = 5.07 μV, *t*(29) = 6.45, *P* < 0.0001), but also during inclusion, albeit to a lesser extent (self = 6.60 μV, other = 4.87 μV, mean difference = 1.73 μV, *t*(29) = 2.20, *P* = 0.036). No other main effects or interactions were observed (all *F*(1,44) < 0.02, all *P* > 0.90; Fig. 2).

### Questionnaire Results

To assess participants’ sense of threatened needs, a 2 × 2 repeated-measures ANOVA was run with the factors social context and skin tone, separately for three measures obtained by the NTQ: Perceived proportion of received throws, reported ostracism, and need-threat. For all three measures, a main effect of social context was observed (all *F*(1,44) > 94.19, all *P* < 0.0001, all ${\eta}_p^2$ > 0.68), confirming that exclusion elicited lower perceived proportion of throws, higher reported ostracism, and increased threatening of needs.

#### Perceived Proportion of Received Throws

For perceived proportion of throws, the main effect of social context interacted with skin tone (*F*(1,44) = 9.16, *P* < 0.01, ${\eta}_p^2$ = 0.17); follow-up *t*-tests revealed that while perceived proportion of throws was higher during inclusion for both the same (mean difference = 24.60%, *t*(44) = 10.57, *P* < 0.0001) and different (mean difference = 14.65%, *t*(44) = 6.29, *P* < 0.0001) conditions, this effect was substantially reduced for those in the different condition. To compare the skin tone conditions more directly, additional follow-up independent-samples *t*-tests revealed lower perceived throws for different participants (28.81%) than same participants (35.33%) during inclusion (mean difference = 6.52%, *t*(87) = 2.66, *P* = 0.018); this skin tone difference was not present during exclusion (mean difference = −3.44%, *t*(87) = 1.40, *P* = 0.164).

#### Reported Ostracism

For reported ostracism, the main effect of social context also interacted with skin tone (*F*(1,44) = 13.02, *P* < 0.001, ${\eta}_p^2$ = 0.23). Follow-up *t*-tests revealed that while reported ostracism was higher during exclusion for both the same (mean difference = 4.46, *t*(44) = 9.41, *P* < 0.0001) and different (mean difference = 2.04, *t*(44) = 4.31, *P* < 0.001) conditions, this effect was substantially reduced for the different condition. Again, to more directly compare skin tone differences, additional independent-sample *t*-tests revealed that during inclusion, there was no skin tone difference in reported ostracism (mean difference = 0.95, *t*(88) = 1.96, *P* = 0.053), though different participants showed a trend toward higher reported ostracism. During exclusion, reported ostracism was significantly lower for different participants (6.75) than same participants (8.21, mean difference = 1.46, *t*(88) = 2.99, *P* < 0.01).

#### Need Threat

For need-threat scores, the same interaction between social context and skin tone was observed (*F*(1,44) = 15.48, *P* < 0.001, ${\eta}_p^2$ = 0.26). Follow-up *t*-tests revealed that while the effect of exclusion (vs. inclusion) on needs was significant for both the same (mean difference = 2.75, *t*(44) = 9.81, *P* < 0.0001) and different (mean difference = 1.19, *t*(44) = 4.24, *P* < 0.001) conditions, this effect was reduced in the different condition. Again, follow-up independent-samples *t*-tests compared skin tone differences, and revealed higher threatening of needs among different participants (3.19) than same participants (2.44) during inclusion (mean difference = 0.75, *t*(85) = 2.39, *P* = 0.019). However, different participants (4.38) were significantly less threatened than same participants (5.19) during exclusion (mean difference = 0.81, *t*(85) = 2.60, *P* = 0.011).

#### Rejection Sensitivity

Finally, an independent-samples *t*-test on rejection sensitivity, as measured by scores on the RSQ, showed no difference between groups (mean difference = 0.33, *t*(46) = 0.40, *P* = 0.69).

### Supplementary Analysis: Order Effects

Because we counterbalanced the order in which participants experienced inclusion and exclusion, this allowed us to compare the pattern of results depending on this order. This was done by including the factor order (included first, excluded first) into the ANOVA model, resulting in a 2 × 2 × 2 × 2 mixed design ANOVA. However, since our power analysis did not account for any interactions with order, we treat this as an exploratory analysis.

For the P3a, possession interacted with order (*F*(1,44) = 17.24, *P* < 0.001, ${\eta}_p^2$ = 0.28). Follow-up *t*-tests indicated no difference between self and other events for those in the included first condition (mean difference = 0.46 μV, *t*(44) = 0.76, *P* = 0.45), but significantly larger responses to self (8.62 μV) versus other (4.55 μV) events for those in the excluded first condition (mean difference = 4.07 μV, *t*(44) = 6.63, *P* < 0.0001). There were no other main effects or interactions involving the factor order (all *F*(1,44) < 2.85, all *P* > 0.09).

For the P3b, social context interacted with order (*F*(1,44) = 12.15, *P* < 0.01, ${\eta}_p^2$ = 0.22), indicating significantly larger responses during exclusion (4.73 μV) than inclusion (6.17 μV) for those excluded first (mean difference = 1.45 μV, *t*(44) = 3.87, *P* < 0.001), but no difference for those included first (mean difference = 0.40 μV, *t*(44) = 1.05, *P* = 0.30). In addition, there was a trend toward a three-way interaction between, possession, social context, and order (*F*(1,44) = 3.51, *P* = 0.068, ${\eta}_p^2$ = 0.07). No other main effects or interactions involving the factor order were significant (all *F*(1,44) < 1.37, all *P* > 0.24).

**Figure 3:**
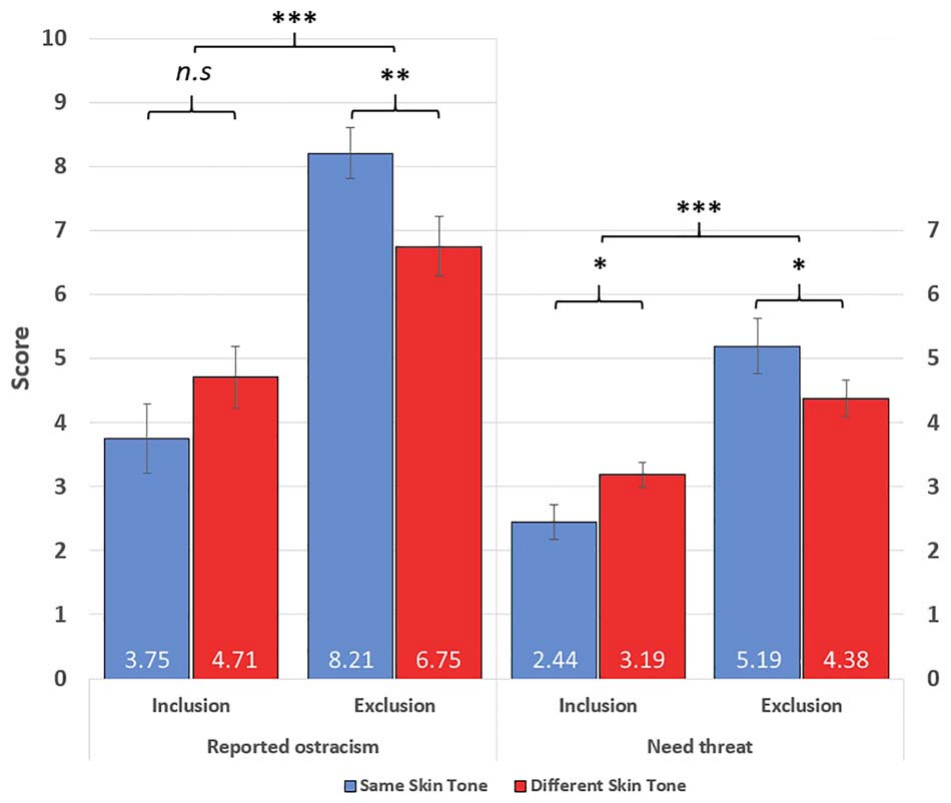
Average scores from the NTQ, depicting reported ostracism and overall need threat (error bars represent SEM). Participants in the same versus different conditions are compared, separately for inclusion and exclusion. Participants in the different condition showed higher threatening of needs than those in the same condition during inclusion, but lower threatening of needs and lower reported ostracism during exclusion.

The questionnaire data were also run with the addition of the factor order. This revealed a larger overall perceived proportion of throws for those in the excluded first group (19.66%) relative to the inclusion first group (24.86%, mean difference = 5.21%, *F*(1,44) = 8.27, *P* < 0.01, ${\eta}_p^2$ = 0.16; Fig. 3).

## Discussion

This study aimed to determine whether perceived racial differences between Cyberball players, as cued by the skin tone of avatars, would modulate expectations of exclusion, as indexed using the P3b component. In particular, we predicted that participants whose avatars had a different skin tone from their coplayers would maintain an expectation of exclusion even while experiencing inclusion. We analyzed P3b mean amplitudes in response to ball throws that took place within periods of inclusion and exclusion. During both periods, these “visual out-group” participants elicited an enhanced P3b when receiving the ball, suggesting that receiving the ball violated their representation of their own participatory status (Gutz et al. 2015). We suggest that this reflects a context-related change in attentional processing, which can be interpreted as the assignment of subjective relevance to this event (Kiat et al. 2018). The mismatch between these participants’ perceived exclusionary status and the inclusionary nature of the game suggests that they expected to be excluded during inclusion, while visual in-group participants did not. Since these groups differed only in terms of the skin tone of the coplayers relative to the participant, and since skin tone was not made explicitly salient or relevant during the game, this group difference suggests that skin tone acts as a subtle cue to activate expectations of exclusion. The lack of any group differences on the pattern of P3a activity highlights that skin tone differences specifically influence cognitive processes related to subjective relevance and the updating of working memory representations, as opposed to changes in the orientation of focal attention (Polich 2007).

Comparisons of P3b amplitudes in response to self and other ball-possession events were used as a marker for the expectation of exclusion. As described already, several previous ERP studies using the Cyberball paradigm (e.g., Gutz et al. 2011; Themanson et al. 2013; White et al. 2013) have shown that during a period of actual exclusion, but not inclusion, participants do indeed show larger P3b responses to self events, and this has been attributed to an expectancy-based account of social participation (Weschke and Niedeggen 2015; Schuck et al. 2018). The current study supports these findings, and provides further support for more recent findings that the P3b response during Cyberball can be modulated without any changes in actual experience. Just as in Gutz et al. (2015), we found different patterns of P3b activation between groups of participants who both underwent the same periods of inclusion and exclusion. While Gutz et al. (2011) demonstrated this effect in relation to clinical psychological factors, more recent work by Kiat et al. (2017) has revealed similar P3b pattern modulations within racial minority participants, driven by subtle social cues in the form of racially stereotyped choices. However, the current study is the first to confirm that this modulation can take place during Cyberball as a sole result of task-irrelevant visual cues, and between two groups who do not differ in trait rejection sensitivity.

An alternative interpretation of differences in P3b amplitude between self and other events is that these events vary in the level of self-relevance and the extent to which they require an upcoming decision (see Kawamoto, Nittono, and Ura 2013). Self events indicate that the participant is involved in the observed event and that a decision is required regarding the next throw, thus necessitating more attention. Other events do neither of these things and do not place the same demands on attention. However, this interpretation does not explain the absence of a significant P3b difference between self and other events during inclusion in the same skin tone condition. Furthermore, differences in the pattern of P3b results across skin tone groups cannot be explained without acknowledging the influence of the avatars’ colors. Therefore, we suggest that some degree of attentional processing in response to self and other events is modified either by skin tone differences, or by social cues that these differences elicit.

Why might skin tone cue exclusionary outcome expectations in this way? Considering the similarity between this paradigm and that of Goodwin et al. (2010), in which coplayer skin tone was also manipulated, it is likely that individuals search for information to which they can attribute exclusion, and this attribution is believed to account for many of the observed changes in psychological responses to exclusion (Crocker and Major 1989; Mendes et al. 2008). In fact, Goodwin et al. provide convincing evidence that attributing exclusion to prejudice is strongly related to subsequent psychological recovery. However, most of these conclusions are drawn on the basis of self-report measures taken after exclusion has occurred. For this reason, it is difficult to determine whether attributions take place after the experience of exclusion, and retrospectively modify the cognitive appraisal of this experience, or whether the cues that are known to drive these attributions (such as skin tone differences) also influence the cognitive processing of exclusion as it occurs. The ERP technique utilized in the current study sheds light on these processes and reveals that, in addition to facilitating retrospective attributions of exclusion to prejudice, subtle visual cues prospectively modify the formation of social expectations as interactions take place in real time.

Assuming that skin tone differences modulate exclusionary expectations in this way, it may be expected that the order of inclusionary and exclusionary periods would have produced a similar effect. Some previous research raises the possibility that the ongoing experience of inclusion and exclusion shapes expectations of upcoming exclusion; for example, exclusionary expectations may increase after having just experienced exclusion relative to having just experienced inclusion. However, ERP evidence for such order effects is not clear: While Gutz et al. (2011) found that the exclusion effect on the P3a was significant only when participants were included first and not when excluded first, no such interaction was observed for the P3b, and to our knowledge no other ERP studies have demonstrated systematic order effects on the P3b in Cyberball paradigms—instead, the majority of ERP studies since Gutz et al. have opted to maintain a fixed order (e.g., Themanson et al. 2015; Niedeggen et al. 2019). As demonstrated by our exploratory analysis of order effects, the P3b exclusion effect trended toward interacting with order. Since our study only manipulated order as a counterbalancing measure and was thus not designed a priori to investigate its influence, we interpret this finding only as a motivation for future research, firstly to continue systematically investigating the influence of temporal order on neurophysiological and psychological responses to inclusion and exclusion, and to further explore the relationship between order and other factors (such as visual group differences) that may cue exclusionary expectations.

Another assumption leading from an expectancy-based account of our results is that responses should change dynamically within a continuous period of Cyberball. Specifically, differences in P3b amplitude between self and other events should reduce as the game continues. This has indeed been observed in recent studies (Schuck et al. 2018; Niedeggen et al. 2019). To investigate this, we ran a separate ANOVA which mirrored the main tree-way ANOVA but also included the within-subjects factor “half,” comprising two levels (first half and second half), with ERP events divided chronologically within the inclusion and exclusion periods (The main effect of half (*F*(1,44) = 13.99, *P* < 0.001, ${\eta}_p^2$ = 0.24) interacted with social context (*F*(1,44) = 5.66, *P* = 0.02, ${\eta}_p^2$ = 0.11) and with possession (*F*(1,44) = 7.91, *P* < 0.01, ${\eta}_p^2$ = 0.15.) The three-way interaction between these factors was not significant (*F*(1,44) = 0.07, *P* = 0.80).). This analysis revealed a general reduction of P3b amplitudes over time, as well as a reduction in the difference between P3b responses to self versus other events. However, the possession × social context interaction, which marked the exclusion effect seen in the main model, did not change throughout the course of each game. This finding is not in line with previous studies, raising questions about the reliability of adaptation effects in Cyberball and highlighting the need for more research to assess the conditions under which expectations might change dynamically. The current study may constitute an example of visual cues creating a priori expectations which are robust in the face of current experience.

The link between skin tone and attribution drawn by Goodwin et al. (2010) mirrors the link between skin tone and the expectation of exclusion observed in the current study. To assess this more closely, we added an additional set of questions half way through data collection (24 participants), for participants to respond to at the very end of the experimental session. An independent-samples *t*-test revealed that attributions (of exclusion) to racism were significantly higher for those in the different condition (mean difference = 2.14, *t*(22) = 7.21, *P* < 0.0001). While this supports our claim that skin tone differences may act as a common cause of both attributions for exclusion and the formation of expectations of exclusion, this should be treated as an exploratory finding that warrants more systematic investigation. In particular, it will be important to investigate how direct the link between attributions and expectation formation is; as there was very little variation among responses in the current study, it was not feasible to directly correlate attribution scores with the magnitude of the P3b effect.

In addition to the ERP data, the questionnaire data revealed a complex but intriguing set of effects of skin tone difference on perceived proportion of throws received, reported ostracism, and threatening of needs. Specifically, during inclusion, visual out-group members reported receiving the ball less often than visual in-group members did, as well as showing a marginal increase in reported ostracism and an increased threatening of needs. These results are in line with previous claims that members of stigmatized groups sometimes exhibit higher threat vigilance and rejection sensitivity and may have an expectation of being excluded (Major et al. 2003; Clark et al. 2006; Carter et al. 2013). Intriguingly, responses during exclusion showed the opposite pattern of results: visual out-group members reported significantly less ostracism and reduced need threat relative to visual in-group members. This is indicative of other research which suggests the presence of cognitive coping mechanisms that can act as a protective buffer to the harmful effects of exclusion (Crocker and Major 1989; Crocker et al. 1991; Cohen et al. 1999). On the basis of our ERP results, one mechanism for this may be the formation of exclusionary expectations. Ultimately, our questionnaire data add to the literature highlighting the complex psychological impact of intergroup exclusion, and suggest that the associated cognitive coping mechanisms may attenuate negative outcomes during actual exclusion, but sustain negative outcomes outside the context of exclusion (Clark et al. 2006; Hawkley and Cacioppo 2011; Hicken et al. 2013, 2014).

While much research highlights the differences between reflexive needs, which tend to be affected equally regardless of factors such as group membership, and reflective needs, which often show reduced recovery after outgroup exclusion (see Wirth and Williams 2009; Goodwin et al. 2010), our study is difficult to interpret within the context of reflexive and reflective needs, since only one questionnaire was taken for each period of inclusion/exclusion. For this reason, we make no claims about the differential impact of exclusion on reflexive versus reflective needs, and instead take our results to suggest that, generally, participants made to appear visually different appear less harmed by exclusion but more negatively affected by inclusion. Importantly, our results demonstrate that these effects can be driven by immediate context, rather than purely by prolonged, lived experience. Because the relative proportion of White and Brown skinned participants was kept constant across skin tone conditions (14 and 10 respectively per condition), our study reveals that even members of nonmarginalized, racial majority groups can experience heightened threat vigilance purely based on observed differences between their own skin tone and that of their interaction partners. These subtle, task-irrelevant visual cues alone are sufficient to trigger changes in the psychological response to exclusion.

To what extent are the results of this study caused by skin tone and its connotations for racial group membership? One possibility that cannot be ruled out is that the observed pattern of P3b results was driven by low-level visual differences entirely unrelated to high-level properties such as group membership. Another alternative possibility is that while skin tone differences created general perceptions of ingroup/outgroup dynamics, they may not necessarily be related to racial prejudice itself. To counter these possibilities, there is some evidence suggesting that the type of identity differences between Cyberball players can influence the psychological responses to exclusion. Wirth and Williams (2009) manipulated either the temporary group membership of participants versus coplayers via avatar colors (green vs. blue), or their permanent group membership via gender. By comparing reflexive and reflective need fulfillment, the authors showed that recovery from the initial harm of exclusion was reduced when participants were identified by permanent versus temporary group membership, indicating that the effect of these visual differences was at least partly due to the higher level social connotations that they evoked. However, research investigating the P3b component during Cyberball has not yet delved into this question, and it remains to be seen whether the P3b modulations observed in the current study could be driven by other forms of visual difference between players. Among other areas of the literature, it is known that minimal group differences can result in changes in behavior and perception toward arbitrarily defined ingroup and outgroup members (Van Bavel et al. 2008), and can even override the effects of race (Van Bavel and Cunningham 2009). To investigate the strength of minimal groups in influencing exclusionary expectation formation within an ERP Cyberball paradigm, manipulating shirt color would provide a cue that is as visually salient as skin tone but without any obvious connotations for permanent group membership such as ethnicity.

In a similar vein, the question arises as to whether group differences must be visible in order to shape expectations about upcoming social interactions. Considering that these differences invoke high-level social concepts (e.g., Mendes et al. 2008; Wirth and Williams 2009), it would be logical to assume that visibility is not a requirement; however, virtually no research (to our knowledge) has directly examined exclusion by nonvisible outgroups, which exist in the real world in countless forms (political affiliation, religious beliefs, socio-economic status, and psychological disorders, to name only a few). A fruitful direction for future research would be to make group differences salient using nonvisual cues such as verbal descriptions.

In conclusion, the current study highlights the power of a simple, task-irrelevant visual cue in influencing the experience and psychological impact of social interactions. In line with the expectancy-based account of the P3b in Cyberball (Kiat et al. 2017, 2018), we suggest that visual skin tone differences between players and interaction partners can activate the expectation to be excluded in preparation for upcoming social interactions. Such expectations of exclusion may be linked to increased vigilance and a process that buffers the effects of exclusion, thus modulating the impact on psychological wellbeing; specifically, vigilance and associated processes may reduce the short-term harm of actual exclusion, but impair need fulfillment outside of exclusionary contexts. Ultimately, this study adds to the literature demonstrating the impact of high-level social information on real-time neural responses to inclusion and exclusion.

## Notes

*Conflict of Interest*: None declared.

## Funding

This work was supported by the Canada Foundation for Innovation, Natural Sciences and Engineering Research Council of Canada, and Social Sciences and Humanities Research Council of Canada.

## Data Availability

All raw data from this study are available directly from the authors upon request.
